# Traditional Chinese medicine on treating chronic prostatitis/chronic pelvic pain syndrome: A systematic review and meta-analysis

**DOI:** 10.1097/MD.0000000000016136

**Published:** 2019-06-28

**Authors:** Yahui Xue, Yunyun Duan, Xiaoyong Gong, Wei Zheng, Yongqiang Li

**Affiliations:** Department of Andrology, The Second Affiliated Hospital of Shaanxi University of Traditional Chinese Medicine, Shaanxi, China.

**Keywords:** chronic pelvic pain syndrome, chronic prostatitis, systematic review, traditional Chinese medicine

## Abstract

**Background:**

Chronic prostatitis/chronic pelvic pain syndrome (CP/CPPS) is a common urinary system disease in the male population. Recent studies have shown that traditional Chinese medicine (TCM) can alleviate the pain caused by CP/CPPS to a certain extent and improve the quality of life of patients. In this systematic review, we aim to evaluate the effectiveness and safety of TCM for chronic prostatitis/chronic pelvic pain syndrome.

**Methods and analysis:**

We will search for PubMed, Cochrane Library, AMED, EMbase, WorldSciNet; Nature, Science online and China Journal Full-text Database (CNKI), China Biomedical Literature CD-ROM Database (CBM), and related randomized controlled trials included in the China Resources Database. The time is limited from the construction of the library to May 2019. The quality of the included randomized controlled trials (RCTs) will be evaluated with the risk of bias (ROB) tool and evidence will be evaluated by Grading of Recommendations Assessment Development and Evaluation (GRADE). STATA 13.0 and Revman 5.3 will be used to perform a systematic review and meta-analysis to synthesize direct and indirect evidence.

**Ethics and dissemination:**

This systematic review will evaluate the efficacy and safety of TCM for treating chronic prostatitis/chronic pelvic pain syndrome. Because all of the data used in this systematic review and meta-analysis has been published, this review does not require ethical approval. Furthermore, all data will be analyzed anonymously during the review process trial.

**Trial registration number:**

PROSPERO CRD42019131527

## 1 Introduction

Chronic prostatitis/chronic pelvic pain syndrome (CP/CPPS), a common urinary system disease in the male population, has the symptoms of repeated, long-term pain and discomfortableness around pelvic floor area and lumbosacral portion, besides, varying degrees of lower urinary tract symptoms (frequent urination, urgency, dysuria, urinary incontinence, etc.) would also become visible.^[1–2]^ Some patients may also suffer from dizziness, memory loss, sexual dysfunction, and even depression.^[3]^ Epidemiological investigations have shown that 2.2% to 13.8% of adult men are suffering from it, and approximately 30% to 50% of men would suffer from it in particular periods of their lives. Related literature reports that the prevalence of CP/CPPS and the related symptoms of it in China is as high as 46.6%.^[4,5]^ Its impacts not only deteriorate the patients’ physical and psychological health, but also the social economy, which, because of the high incidence and the recurrence. Thus, the National Institutes of Health (NIH) has listed CP as one of the chronic diseases that affect the quality of life of residents, which include myocardial infarction, unstable angina, and active Crohn disease.^[6]^

The pathogenesis of CP/CPPS is complicated which has not yet been completely illuminated. Literature reports that there are kind of connections between prostate hyperplasia and CP/CPPS.^[7,8]^ At present, there is no specific therapy for this disease in modern medicine and we mainly adopt antibiotics, α-blockers, etc., however, multicenter clinical studies have shown that the curative effect of single treatment mode is limited or even invalid.^[9–11]^ In addition, due to the specificity of the anatomical structure of prostate—the deeper intima of the capsule, it is easy to cause local microcirculation disturbance, blockage of blood stasis, drainage blocked. All the factors above make the disease become recurrent and chronic.^[12]^

As the most important part of Chinese medicine, Chinese herbal medicine has been widely used in clinical trials of CP/CPPS in recent years. TCM believes that the cause of CP/CPPS is mainly in the kidney.^[13,14]^ Yin deficiency and heat, qi stagnation and blood stasis is its main pathogenic factor. Through the application of traditional Chinese medicine in the treatment of CP/CPPS's unique diagnosis and treatment system, clinical efficacy is significant.^[15,16]^ Modern research has shown that effective active ingredients in traditional Chinese medicine can improve the blood supply of peripheral blood vessels and achieve therapeutic purposes.^[17,18]^ Through the action mechanism of multifaceted and multitarget, TCM regulates the body function as a whole and has unique advantages in the treatment of CP/CPPS. After preliminary search and analysis of database, we found that the frequency of randomized controlled trials of TCM treatment in CP/CPPS has been showing an increasing trend.^[19]^ Previous clinical trials have shown that TCM could ameliorate pain and improve the quality of lives in patients who suffer from CP/CPPS, and these effects are sustained.^[17,20]^ However, due to the limitation of the scale and sample size of the clinical centers, the current level of evidence-based medical evidence is still not sufficient. Therefore, we hope to evaluate the efficacy and safety of TCM in treating CP/CPPS by using network meta-analysis, which aim to provide sufficient evidence for its clinical application.

## 2 Methods

This systematic review protocol has been registered on PROSPERO as CRD42019131527. (http://www.crd.york.ac.uk/PROSPERO/display_record.php?ID=CRD42019131527). The protocol follows the Cochrane Handbook for Systematic Reviews of Interventions and the Preferred Reporting Items for Systematic Reviews and Meta-Analysis Protocol (PRISMA-P) statement guidelines. We will describe the changes in our full review if needed.

### 2.1 Inclusion criteria

#### 2.1.1 Types of studies

This study will include all the RCTs that relate to TCM therapy in treating CP/CPPS. For the included trials, the investigators need to precisely report the stochastic methods, TCM treatment details and parameters, diagnostic criteria, and efficacy evaluation they based on. No limitation to whether it is published or not. The experiment is limited to humans. Language is limited to Chinese and English.

#### 2.1.2 Types of participants

Male patients who were definitely diagnosed with CP/CPPS would be included (refer to the National Institutes of Health diagnostic criteria for CP/CPPS expert consultation). The cases which relate to prostatic hyperplasia, prostate cancer or other prostate-related diseases would be excluded. In addition, there are no limitation in region, citizenship, nationality, and source of cases.

#### 2.1.3 Types of interventions

##### 2.1.3.1 Experimental interventions

The drug composition, the dose-specific Chinese medicine preparation, or the combined western medicine are used as experimental interventions. Both prescription and Chinese patent medicines will be included. Other traditional Chinese medicine treatments such as intravenous medication, acupuncture, and massage will be limited.

##### 2.1.3.2 Control interventions

As for the control interventions, who accepted simple western medicine can be used as a control intervention or didn’t get any treatment as a blank control would be adopted. However, once they had accepted the therapy of TCM, the trials will be rejected.

#### 2.1.4 Types of outcome measures

##### 2.1.4.1 Primary outcomes

The National Institutes of Health's Symptom Score Index (NIH-CPSI) score for CP/CPPS will be used as the primary outcome measure. Every dimensions (degree of pain, urinary symptoms, quality of life) of NIH-CPSI will be evaluated, and the lower the total score, the lighter the clinical symptoms of the patient.

##### 2.1.4.2 Secondary outcomes

Secondary outcomes include: changes in international prostate symptom scores (IPSS) before and after treatment; changes in international erectile function scores (IIEF-5) before and after treatment; comparison of effective rates between groups and the incidence of adverse events.

### 2.2 Data source

Database Search: PubMed, Cochrane Library, AMED, EMbase, World SciNet, Nature, Science online and China National Knowledge Infrastructure (CNKI), China Biomedical Literature CD-ROM Database (CBM), China Resources Database. Search for clinical research literature on TCM CP/CPPS published in domestic and foreign biomedical journals from the establishment of the library to May 2019. Based on the standards of the Cochrane Collaboration Workbook of the International Evidence-Based Medicine Center, a manual and computer-based method will be used to conduct related literature searches. The search terms include: chronic prostatitis, chronic pelvic floor pain syndrome, non-bacterial prostatitis, Chinese medicine, traditional Chinese medicine, proprietary Chinese medicine, Chinese herbal medicine. Manually search for topics, abstracts, etc. related to the research of Chinese Journal of Male Science, Chinese TCM, and TCM. The complete PubMed search strategy is summarized in Table 1.

**Table 1:**
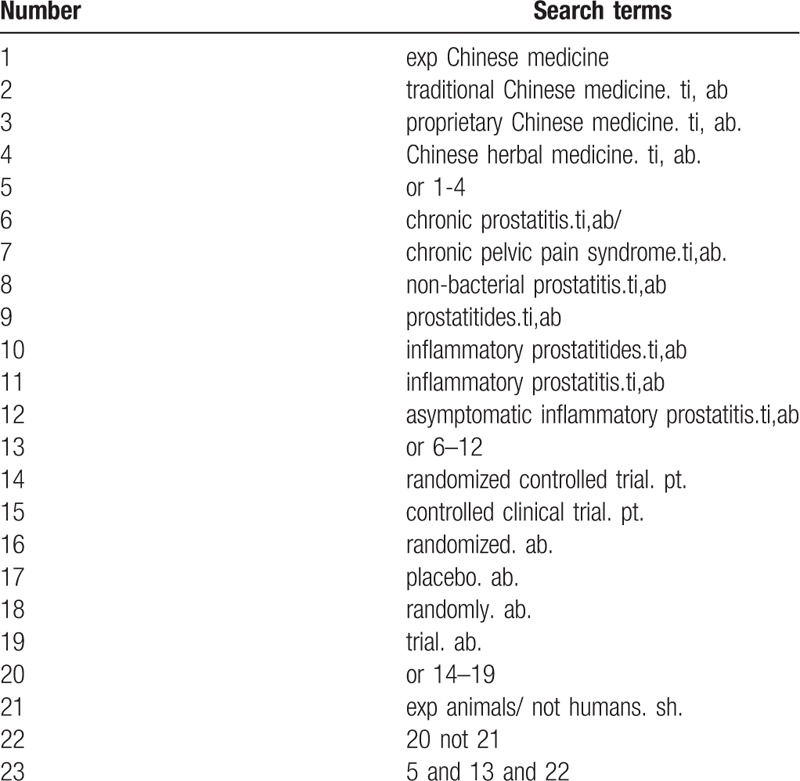
Search strategy used in PubMed database.

### 2.3 Data collection and analysis

#### 2.3.1 Selection of studies

Two investigators used EndnoteX7 software to conduct a preliminary assessment of the title and abstract of each document in the database based on the established criteria for inclusion in the study to select eligible studies. After a preliminary assessment, the full text of the selected literature would be evaluated, and the uncontrolled study, no randomization, inconsistent evaluation criteria, and similar data would be excluded. Any differences in screening that occurred during the screening study would be discussed in order to get consensus, if it still cannot be resolved, then the third author would be intervened.

#### 2.3.2 Data extraction and management

**Figure 1:**
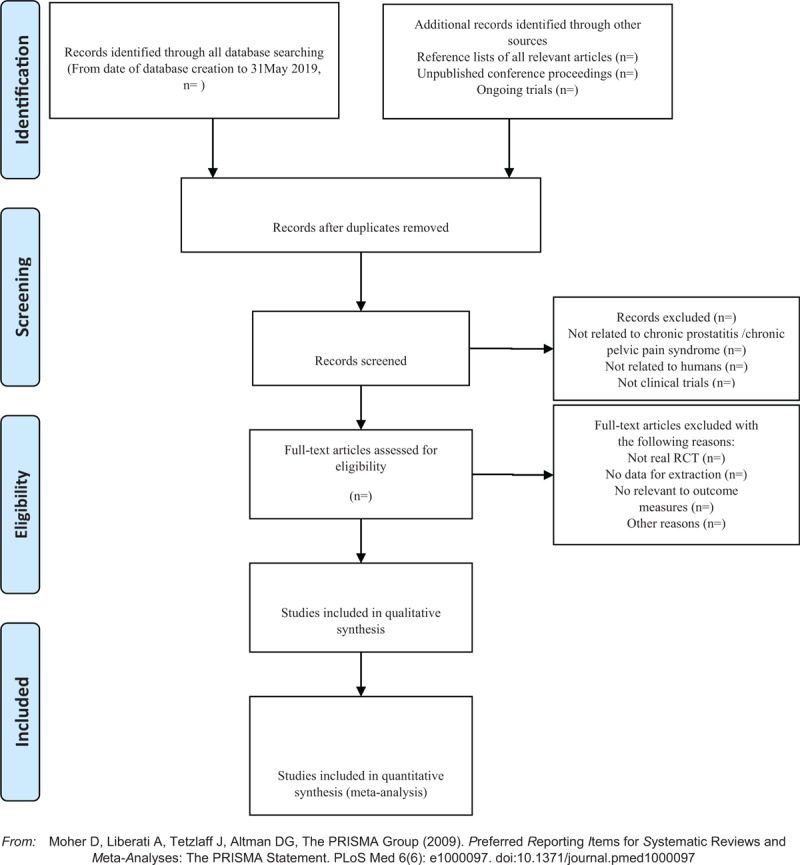
The PRISMA flow chart. PRISMA = Preferred Reporting Items for Systematic Reviews and Meta-Analysis.

Two investigators independently extracted information from the included literature. The extracted content includes research design, random hiding and blinding, basic information of the included cases, intervention methods, observation indicators, and test results of the treatment group and the control group. The extracted literature data will be filled in a unified data statistics table. For studies that provide baseline and posttreatment data, we will estimate the change values by the method recommended by Cochrane. The details of selection process will be shown in the PRISMA flow chart (Fig. 1).

#### 2.3.3 Assessment of risk of bias in included studies

Two investigators will independently evaluate the methodological quality of the included literature by using the Cochrane Collaboration ROB tool which includes whether the random method is correct, whether blinding is used, whether it is hidden, whether it is lost or quit, whether it uses Intent-To-Treat (ITT) analysis, whether the data results are accurate, and other risks of bias. According to the relevant standards in the Cochrane Intervention System Evaluation Manual, it will be divided into low risk, high risk, and unclear.

#### 2.3.4 Dealing with missing data

In the event of data loss during the screening and extraction of literature data, first, we will actively investigate the cause of data loss. Then we will contact the experimental research author by telephone, mail, etc. to achieve the purpose of supplementing the missing data. If the lost data cannot be retrieved, we will only extract and analyze the useful data, besides, we will indicate the situation.

#### 2.3.5 Statistical analysis

The numerical variable will be expressed as the normalized mean difference (SMD) with a confidence interval (CI) of 95%. The heterogeneity of each pairwise comparison will be tested by chi-square test (test level *α* = 0.1). If there is no heterogeneity, a fixed effect model will be used. If there is significant heterogeneity between a group of studies, we will explore the reasons for the existence of heterogeneity from various aspects such as the characteristics of the subjects and the degree of variation of the interventions. Sensitivity analysis or meta-regression and subgroup analysis to explore possible sources of heterogeneity when necessary. We will use qualitative analysis of the funnel plot and graph symmetry to assess publication bias. Quantitative methods such as Begg testing and Egger testing will be used to help assess publication bias in the application.

#### 2.3.6 Assessment of heterogeneity

If there is significant heterogeneity between a group of studies, we will explore the reasons for the existence of heterogeneity from various aspects such as the characteristics of the subjects and the degree of variation of the interventions. Sensitivity analysis or subgroup analysis is performed as necessary to explain heterogeneity.

#### 2.3.7 Assessment of publication bias

The forest map and funnel plot were drawn and analyzed using Rev Man5.3 software, and the funnel plot was used to analyze potential publication bias.

#### 2.3.8 Grading the quality of evidence

The quality of evidence for the main outcomes will also be assessed with the GRADE approach. The evaluation included bias risk; heterogeneity; indirectness; imprecision; publication bias. And each level of evidence will be made “very low,” “low,” “moderate,” or “high” judgment.

## 3 Discussion

At present, there are vast of therapies in treating CP/CPPS, however, the efficacy is still unsatisfactory due to the particularity of the anatomical structure of prostate.^[21]^ Studies have shown that drug intervention can improve the overall NIH-CPSI score and ameliorate most of the symptoms of CP/CPPS patients to a certain extent, but there is no single drug can continue to significantly ameliorate all symptoms of CP/CPPS patients.^[22,23]^ TCM has a profound theoretical foundation and abundant clinical experience in the treatment of CP/CPPS.^[24]^ TCM therapy mainly achieves therapeutic effects by stimulating the body's righteousness and regulating the balance of qi and blood.^[25]^ In recent years, TCM therapy has been widely used in clinical trials of CP/CPPS. Recent studies have shown that TCM can alleviate the pain caused by CP/CPPS to a certain extent and improve the quality of lives of patients.^[26]^

Although abundant studies have evaluated the effectiveness of TCM in treating CP/CPPS, evaluation and comparison between various treatments are still insufficient. To the best of our knowledge, a systematic review and meta-analysis has not been used in recent years to compare the effectiveness of TCM in the treatment of CP/CPPS. The results of meta-analysis can provide a possible ranking for TCM treatment of CP/CPPS. We hope that the results will provide clinicians with the best options for treating CP/CPPS and provide them with research directions. Due to the limited number of relevant high-quality studies and the few sample size included, the strength of the arguments of the conclusions is to some degree limited. Therefore, we hope that more large-scale, high-quality randomized controlled trials should be necessary in the future. Besides, improving the quality of the original research and conducting high-quality multicenter randomized controlled trials to explore the clinical efficacy of TCM treatment of CP/CPPS is also indispensable, through which could make the conclusion more objective and reasonable.

## Author contributions

**Conceptualization:** Yahui Xue.

**Data curation:** Yahui Xue, Yongqiang Li.

**Formal analysis:** Yahui Xue, Yunyun Duan.

**Funding acquisition:** Yunyun Duan, Yongqiang Li.

**Supervision:** Xiaoyong Gong, Wei Zheng.

**Visualization:** Xiaoyong Gong.

**Validation:** Wei Zheng.

**Project administration:** Yongqiang Li

**Writing – review & editing:** Wei Zheng.

**Writing – original draft:** Yongqiang Li.
